# Reply to Confalonieri et al. Comment on “Colombo et al. Managing Retinitis Pigmentosa: A Literature Review of Current Non-Surgical Approaches. *J. Clin. Med.* 2025, *14*, 330”

**DOI:** 10.3390/jcm14082557

**Published:** 2025-04-08

**Authors:** Leonardo Colombo, Jacopo Baldesi, Salvatore Martella, Chiara Quisisana, Aleksei Antico, Luca Mapelli, Stefania Montagner, Alberto Primon, Luca Rossetti

**Affiliations:** 1 Eye Clinic, ASST Santi Paolo e Carlo Hospital, University of Milan, 20142 Milan, Italy; jacopo.baldesi@unimi.it (J.B.); salvatore.martella@unimi.it (S.M.); chiara.quisisana@unimi.it (C.Q.); alekseiantico110@gmail.com (A.A.); lmapelli1424@gmail.com (L.M.); luca.rossetti@unimi.it (L.R.); 2 Eye & Vision—Visual Rehabilitation Center, 20128 Milan, Italy; stefania.montagner@eyevision.it (S.M.); alberto.primon@eyevision.it (A.P.)

We would like to express our sincere gratitude to the authors of the comment [1] for taking the time to read our article, *Managing Retinitis Pigmentosa: A Literature Review of Current Non-Surgical Approaches* [2], and for providing us with their detailed feedback. We appreciate the recognition of the importance of a holistic approach and the need to consider non-surgical interventions in the management of patients with retinitis pigmentosa (RP).

However, we feel it is necessary to specifically address several points of divergence between our two papers. Although both our reviews focus on RP, we believe that the focus of the two articles is significantly different to warrant separate treatments as all the other peers who have examined it confirmed. Our article specifically highlights established non-surgical interventions—such as visual aids, photoprotection, and psychological counselling—which play a crucial role in maintaining the quality of life of patients with RP. These interventions, although well recognized, remain underused or underappreciated in clinical practice and our review aims to emphasize their importance in the absence of curative therapies.

Differently, the work of Confalonieri et al. places considerable emphasis on emerging therapies, such as gene therapy, mesenchymal cell-based approaches, and other novel interventions. While we acknowledge the importance of these developments, which will undoubtedly shape the future of treatments for RP and other retinal dystrophies, we chose not to delve into them in our review. Our intention was to explore what is currently feasible in the management of patients awaiting the approval of new therapies other than voretigene neparvovec. The different perspectives of the two articles appear to us to be their strength as well and one of the reasons to maintain them separate—also, not last, for a matter of clearness providing the readers with the chance to deepen such different areas of investigation.

Our 40+ years of experience as a reference center for RP, highly specialized national and international collaborations (e.g., the European ERN-EYE network, of which our center is a member), and our ongoing engagement with patient associations have been key factors in crafting an updated review based on the topics discussed in our article. We have emphasized in our work that the evidence supporting these interventions is often limited and needs further research.

Furthermore, due to the nature of the review, some topics have clearly already been covered in previous publications. Therefore, the novelty of our approach lies in the contextual discussion and the idea that each intervention, individually and especially synergistically, can represent a valuable means of improving the quality of life for RP patients.

These initial considerations highlight that neither the overlap nor the inclusion of the mentioned review (as well as others) seem necessary or appropriate in the context of our work. This would shift the focus away from the central theme we have sought to address. Our focus on non-surgical management was intended to serve a distinct purpose, which does not align with a broader discussion of experimental therapies.

In conclusion, we value the ongoing debate in the field of RP research and trust that future publications will progress through meaningful and insightful collaborations, drawing also on distinct perspectives such as those offered by our reviews for the benefit of patients today and in the near future.
